# Supplementary material for: Are carbon emissions trading and green financial instruments synergistic? -Comprehensive quantitative research based on content analysis
Source: PLoS One. 2024 Mar 7;19(3):e0298601. doi: 10.1371/journal.pone.0298601 (PMC10919601; doi:10.1371/journal.pone.0298601)
Supplement: S1 Data — (ZIP) [file pone.0298601.s001.zip › Data/地级市Z_F工作报告.zip!/░▓╗╒/╬▀║■╩╨/└┤╫╘╣1⁄2╓┌║┼í╛┬φ┐╦╩2╛▌═°í┐_2009.docx]

——2009年 1月5日在芜湖市第十四届人民代表大会第三次会议上

市长 杨敬农

各位代表：

现在，我代表市人民政府，向大会作政府工作报告，请予审议，请各位政协委员和其他列席人员提出意见。

一、2008年工作回顾

过去的一年是极不寻常、极不平凡的一年。这一年喜事多，胡锦涛总书记年初亲临芜湖视察，为芜湖发展指明了前进方向；改革开放30周年之际，我市被列入在中国特色社会主义旗帜指引下开拓成功发展之路的全国18个典型地区。这一年难事多，罕见冰雪灾害突如其来，国际金融危机愈演愈烈。一年来，在中共芜湖市委的正确领导下，全市上下深入贯彻落实科学发展观，锐意进取，攻坚克难，全面完成了市十四届人大一次会议确定的主要目标任务，国民经济呈现增长较快、效益较好、物价回稳、结构优化、民生改善的良好态势，各项事业全面发展，社会和谐稳定。

2008年，全市实现生产总值745亿元（预计数，下同），比上年增长16%，为10年来第二个高增长年，一、二、三次产业比重由上年的5.8∶57∶37.2调整为5.1∶60.7∶34.2。全社会固定资产投资615亿元，增长41.1%。财政收入123.85亿元，增长17.8%，其中地方财政收入53亿元，增长20.7%。城市居民人均可支配收入14680元，增长10.9%；农民人均纯收入5800元，增长11.4%。城镇新增就业岗位5.49万个，城镇登记失业率控制在3.95%以内。人口出生率控制在9‰以内。万元地区生产总值综合能耗下降5%，二氧化硫、化学需氧量分别减排1.5%和1%。

一年来，我们主要做了九个方面工作：

第一，着力加强经济运行调节。过去一年，宏观经济环境复杂多变，百年不遇的国际金融危机对经济运行形成巨大冲击。为力保经济增长，我们认真贯彻中央和省委、省政府决策部署，紧密结合芜湖实际，见事早，判断准，行动快，及时果断地采取了一系列应对措施，取得了积极成效。一是加强价格调控。面对年初物价过快上涨等问题，我们加强市场监管，实行临时价格干预，落实城市公交、出租车油价、农村客运和供水价格补贴共计6871万元，建立粮油等重要商品储备制度，物价过快上涨势头得到有效遏制。二是稳定房地产市场。我们在全国较早出台了《关于促进我市房地产市场持续健康发展的若干意见》，财政安排补贴资金4000万元，改善低收入人群住房条件，增强消费者和投资者信心。全年房地产开发完成投资143亿元，增长60.9%；市区商品房批准销售200万平方米，增长42.6%；市区商品住宅销售全年均价每平方米4050元，比上年回落4.2%；安置房开工268万平方米、竣工161万平方米。三是增强企业竞争力。年初我们以1号文件形式出台了《关于大力提高自主创新能力加快产业结构优化升级的若干意见》及28项配套措施，财政安排4.8亿元，推动企业提高研发能力。进入下半年，全球金融危机对我市经济的影响逐步显现，我们超前谋划，积极应对，9月份制定了《关于进一步优化环境促进中小企业又好又快发展的若干意见》，财政安排2.9亿元，鼓励中小企业加快发展上台阶。四是强化政府服务。成立了经济运行调度领导小组，建立了市领导联系企业制度，组成多个工作组分赴企业，协调解决相关问题。建立了中小企业担保保费补贴和担保机构担保风险补偿机制，财政安排8500万元支持担保体系建设。通过开展银企对接活动、创新金融产品等方式，帮助企业解决融资困难，签订授信合作协议金额达230亿元。降低企业运营成本，清理行政事业性收费和经营服务性收费，取消、暂停收费项目和降低收费标准85项，年减轻社会负担1.43亿元；下调部分社会保险缴费费率，年减轻社会负担1.5亿元。五是推进重大项目建设。奇瑞轿车三厂、瑞江改装车二期、荻港海螺水泥三期、新兴铸管锻造坯料生产线、华电芜湖电厂一期、海螺余热发电设备等项目建成投产，中达电子、响水涧抽水蓄能电站等项目建设步伐加快，奇瑞轿车五厂、芜湖电厂五期、信义环保特种玻璃、宝成鞋业、芜雁高速、芜申运河、宁安城际铁路等项目开工建设。六是抢抓国家扩大内需政策机遇。积极争取了上级专项资金21亿元，同比增长45%。谋划新上了一批增强发展后劲的重大项目，核电项目取得重大进展，京福、合芜杭、皖赣客运专线，皖赣、淮南、宁铜线铁路电气化改造和芜湖火车站扩建项目列入铁道部建设计划。通过不懈努力，全市经济保持了较快增长，规模以上工业实现增加值371亿元，增长21.3%。全市规模以上工业企业1148户，净增274户。汽车及零部件、材料、电子电器三大支柱产业进一步壮大，实现总产值930亿元，增长24%；装备制造业实现产值127亿元，增长80%，成为我市工业第四个超百亿元的产业。全年社会消费品零售总额突破200亿元，达204亿元，增长23%，增幅创近10年来新高，出现了农村消费增长快于城市的良好局面。

第二，着力转变经济发展方式。全面启动合芜蚌自主创新综合配套改革试验区工作，制定了实施方案和配套政策。芜湖汽车电子及关键零部件企业孵化器1号中试厂房投入使用，国家汽车零部件产品质检中心基本建成，奇瑞公司汽车节能环保国家工程实验室开工建设，新建科技企业孵化器6万平方米。新增省级重点实验室4家、省级企业技术中心5家，争取国家、省科技项目75个。145家企业通过高新技术企业评审，奇瑞公司成为首批国家创新型企业。我市11个产品获省名牌称号，申请专利2500件，授权专利1000件，被批准为国家知识产权示范城市创建市。开发复垦整理土地9550亩，耕地总量保持动态平衡。出台了国有划拨土地有偿使用办法，出让经营性用地和工业用地679.3公顷。对市区1999年以来出让的经营性用地进行了全面清查，依法处置闲置土地138.4公顷。南陵县成为全省首批国土资源执法模范县。启动了省节能装备制造基地建设，海螺余热发电等32个节能、资源综合利用项目建设进展顺利。实施了63个减排项目，关停了13家小水泥企业。市区3个污水处理厂建设加快，三县污水处理厂开工建设。强化饮用水源地保护和烟尘治理，清除珍珠养殖水面近3万亩。推进南陵县、芜湖县、弋江区三个全国生态示范区建设，完成了全市污染源普查和“百镇千村万户生态示范工程”目标任务。

第三，着力提升服务业发展水平。奇瑞汽车滚装码头、海螺万吨级件杂货码头、港储10万标箱集装箱码头基本建成，安得物流中心等一批物流项目进展顺利。欧尚超市建成开业，联盛商业广场、美食街二期、小九华商业街、果品冻品海鲜批发市场、小商品批发市场等一批大型商贸项目正积极推进。弋江区禹王宫社区被评为全国商业示范社区。方特欢乐世界主题公园成为长三角旅游新热点，全年接待游客185万人次，有力拉动了相关服务业发展；侨鸿酒店建成开业，碧桂园、新百、阳光半岛等星级酒店建设加快；我市被列入省旅游发展三大中心城市，全年旅游总收入49亿元，增长28%，增幅为5年来最高。成功举办了科博会、汽博会、房博会等24个会展，会展经济效应进一步显现。金融体系建设取得新进展，成立了邮政储蓄银行芜湖市分行，招商银行和6家域外保险机构来芜设立分支机构，浦发行芜湖支行升格为一级分行，扬子农村商业银行在三县布点。妥善处置了农村合作金融机构不良资产2.3亿元，繁昌、芜湖县农村合作银行组建工作进展顺利。完成了金财典当行增资扩股，成立了民强担保公司，全市中小企业担保、典当机构分别达103家、10家，分别新增13家、3家。批准组建小额贷款公司6家。安徽高新技术企业产权交易所挂牌营业。全市金融机构本外币存贷款余额分别达693亿元、563亿元，分别比年初新增99.4亿元、107亿元，分别增长23.7%、24.8%，占全省的比重分别达6.8%、10.4%。域外金融机构在我市贷款余额106亿元，净增22亿元。公用企事业缴费平台基本建成，有效缓解了群众缴费难问题。

第四，着力统筹城乡发展。农业生产全面丰收，实现农林牧渔业增加值38亿元，增长4.5%。全年粮食总产近80万吨，连续五年增产。袁隆平超级杂交稻“种三产四”丰产工程实施面积20.9万亩，平均亩产625公斤，每亩增收170元。新增省级农业产业化龙头企业6家、无公害农产品认证7个、绿色食品认证30个，培育农村合作经济组织285个。认真落实强农惠农政策，全年发放涉农补贴资金1.66亿元。实施农村劳动力转移“阳光工程”，培训农民1.5万人。公共服务和基础设施向农村延伸覆盖成效明显，我市被列入省城乡一体化综合配套改革试验区。探索建立城乡接轨的养老、医疗保障制度。投入2530万元加强中小学标准化建设，南陵一中新校区项目开工。建成了83个村农家书屋，镇、街道文化站建设达标率达40%，在全省率先实现“电影放映村村通”。新增31个行政村通公交班车，基本实现全市村村通公交班车。新增出租车474辆，更换公交车137辆，三山区公交车班线开通，城乡老年人免费在市区乘坐公交车实现同城同待遇，有效缓解了居民出行难问题。芜南路拓宽改造、芜屯路快速通道雨污水管和慢车道工程基本建成，新建农村公路118公里。芜南供水工程进展顺利。14座病险水库除险加固、482公里万亩以上圩口堤防达标建设任务全面完成。市级财政调度资金20.5亿元，有力支持了县区发展。

第五，着力深化改革开放。深入实施创业富民战略，非公有制经济健康发展，规模以上工业企业户数占全市总数的94%，其中全省200强企业51户；非公有制经济增加值占全市经济总量的比重达65%，位居全省第一。加强了国有企业资产监管，市属国有企业资产194.6亿元，比上年增加15.3亿元。深化农村土地承包经营权流转改革，全市流转总面积8.7万亩，净增3.95万亩。积极推进林权制度改革，完成了确权勘界主体改革工作。创新政府管理体制，强化城市建设管理，成立了城乡建设委员会和重点工程建设管理局；调整经济技术开发区机构设置，建立了以聘用制度、目标管理和绩效考核为核心的用人机制，增强了开发区发展活力。市、区事业单位分类改革基本完成，市属事业单位由226个减少到130个，编制由10215人减少到8930人。在全国率先实施医药分开改革，成立了市医疗机构药品管理中心，对市属8家医疗机构实行了药品采购配送统一管理。积极实施惠民直达工程，惠民资金实行“一卡通”发放。企业直接融资取得新进展，海螺水泥、鑫科材料增发募集资金119亿元，占全省的67.4%；奇瑞汽车等3家企业上市申报材料已上报中国证监会。主动参与泛长三角发展分工与合作，举办了承接上海产业转移推介会等12场投资推介活动，积极推进小分队招商工作，奇瑞量子汽车、韩国浦项制铁等一批大项目落户芜湖，新增4家世界500强企业来芜投资，全年实际利用外资5.5亿美元，增长18.8%；实际利用内资435亿元，增长47%。建成了经济技术开发区东区投资服务中心，完成了出口加工区扩区、封关二期工程。全年进出口总额20.4亿美元，增长21.2%，其中出口14.8亿美元，增长20.2%。港口货物年吞吐量5400万吨，增长15.7%。外经、外事、外宣、对台、侨务工作全面推进，与美国西克尔汶市建立了友城关系，对外交往日益扩大。

第六，着力推进城市大建设。去年城市建设政府投资85亿元，新建、续建道路20条，改扩建道路7条，改造背街背巷80条，这是我市有史以来建设力度最大的一年。滨江南路一期、三环路中段延伸段、龙湖路一期建成通车，弋江路、峨山路改扩建和长江路“刚改柔”完成主车道工程，滨江南路二期、利民东路、花津南路、纬五路东段、赭山路“刚改柔”、弋江路南延线等道路工程开工建设。城东新区建设加快，主干路网基本形成。完成保兴垾综合整治清淤和部分景点工程，滨江公园一期向市民开放。启动了三年大绿化工程，新增绿化面积50万平方米。加强了城市生活垃圾无害化处理，垃圾焚烧热电厂扩能一期建成。新建天然气加气站6座。杨家门水厂扩建工程开工。市科技馆、文化馆、图书馆新馆建成开放，新华文化创意产业园、徽派建筑艺术博物馆等项目基本建成，华强文化科技产业园、世茂文化艺术中心、芜湖和瑞动漫科技产业园开工建设。新建了市儿童福利院和救助管理站。加大拆迁工作力度，共拆迁322万平方米。强力推进查处违法建设工作，拆除市区违法建筑50多万平方米。编制了综合交通、商业网点等专业专项规划和一批控制性详规，新一轮城市总体规划获省政府批准。初步建成了数字化城管信息平台系统，城市管理进一步加强。

第七，着力实施民生工程。全年投入25.37亿元，完成了24项民生工程年度目标任务，惠及180万城乡居民。完善了居民最低生活保障制度，全市低保对象实现了应保尽保。农村“五保户”集中和分散供养标准分别为2400元、1800元，分别提高了300元、200元。完善城镇未参保集体企业退休人员基本生活保障机制，提高了5053名未参保人员养老保障水平，人均每月增加260元。新型农村合作医疗参合人数97.8万人，参合率达94.6%。完善城镇居民基本医疗保障制度，参保人数62.3万人。提高城乡医疗救助水平，救助病种由9种扩大到13种，救助对象扩大到所有城乡困难群众。建立了重大传染病病人医疗救治及生活救助保障机制，救治艾滋病人、结核病人、晚期血吸虫病人474例。完善城乡医疗卫生服务体系，投入5000万元支持市二院门诊住院楼、中医院新院区等项目建设，建成镇卫生院、村卫生室、社区卫生服务机构163个。全面实施城乡义务教育经费保障机制改革，免除了21.7万名义务教育阶段学生学杂费，免费为17.2万名农村义务教育阶段学生提供教科书，补助了1685名农村贫困寄宿生生活费。消除了4.3万平方米农村中小学现有D级危房。解决了13万农村人口饮水安全问题。对2880名农村计划生育奖扶对象进行了奖励。为1713名水库移民发放了生活补助。资助高职、中职、技工类家庭困难学生5.9万人次。对432名贫困白内障患者实施了复明手术。完成81个自然村广播电视盲村“村村通”建设任务。廉租住房建设开工13万平方米、竣工9万平方米，对8144户城市低收入住房困难家庭发放租金补贴2324万元。建成 “村村通” 公路延伸工程35公里。实施食品药品安全工程，建立了10个蔬菜检测点，定期对液态奶等12类食品进行评价性检测。市区棚户区改造工程完成拆迁39.3万平方米。完成了市区环境综合整治年度目标任务。实施道路畅通工程，改造拓宽平交道口15处，改造城市干道港湾式公交停靠站台65座、农村公路危桥22座，建成农村道路附属工程1568公里、农村客运站5座、候车亭108座。实施医药分开改革，规范医药购销行为，让利群众1200万元。对2543名城乡就业困难人员实行了托底就业。

第八，着力发展各项社会事业。认真贯彻《就业促进法》，积极开展统筹城乡就业国家试点，全年发放小额担保贷款2.2亿元。实行社会保险 “一票多费”、“五险合一”、“双基数”征缴模式，全市城镇职工养老、失业、医疗、工伤、生育保险参保人数分别比上年净增3.9万人、1.5万人、5.5万人、3.8万人、4.8万人。退休人员养老金月人均由912元提高到1016元，被征地农民养老保障补贴月人均由110元提高到158元。缴存住房公积金的非公有制企业新增112户、职工2.3万人。高中阶段毛入学率达69.1%。新设3所民办中等职业学校。完成市老年大学新教学楼主体工程。新增2家民营医院，建成市医疗废物集中处理中心，弋矶山医院、市四院等基础设施项目进展顺利。举办了央视“激情广场”、市文化艺术节等群众性文化活动。繁昌县成为全国首批文化共享工程示范县。启动了第二次全国经济普查、第三次全国文物普查和非物质文化遗产普查。顺利完成了2008年北京奥运会火炬接力芜湖站传递活动。周吕鑫在奥运会男子十米跳台跳水比赛中勇夺银牌，实现我市运动员奥运奖牌零的突破。加大文明创建力度，我市被评为省首届文明城市、全国未成年人思想道德建设工作先进城市，市慈善总会、奇瑞公司、信义集团以及楚江集团董事长姜纯获中华慈善奖。完成了第七届村委会换届选举。建立了关爱留守儿童长效工作机制。国防后备力量建设进一步加强，人防指挥中心建成。民族、宗教、档案、地方志、人口和计划生育、老龄、残疾人等事业取得新进展。扎实开展安全生产“隐患治理年”活动，排查整治各类隐患391处。及时筛查救治食用三聚氰胺“问题”奶粉患儿，有效防控了儿童手足口病疫情。全面推进治安防控体系建设，社会治安综合治理得到加强，镜湖区通过国家平安城市建设先进县区检查验收。

第九，着力强化政府自身建设。自觉接受市人大及其常委会监督和市政协民主监督，认真执行人大及其常委会决议，办理人大议案和建议119件，政协提案443件，办复率100%。加强与工会、共青团、妇联等社会团体的联系，认真听取各方面的意见和建议。制定了市政府工作规则，在全省率先开展决策咨询课题社会招标工作，完善了政府决策制度。贯彻《国务院关于加强市县人民政府依法行政的决定》，减少和下放行政许可事项57项。建立行政审批电子监察系统，强化市行政服务中心功能，优化了行政审批服务。强化政府性投资项目管理和审计监督，完成项目招标2450个，节约资金7.35亿元。全面贯彻《政府信息公开条例》，积极推进政务公开。加快电子政务建设，网上办公向县区延伸。加强财政支出管理，市直机关支出比预算减少1500万元。加强信访工作，完善党政领导接待日制度，建立信访社情分析研判机制，开展“人民调解百日会战”活动，加大农民工法律援助力度，有效化解了各类矛盾纠纷。扎实开展反腐倡廉制度建设推进年活动，惩治和预防腐败体系建设进一步加强。

各位代表，过去一年，一系列重大突发事件相继发生，面对年初历史罕见的冰雪灾害，江城人民紧急动员，众志成城，携手同心，奋力抗灾，最大限度减少了灾害损失，奏响了战冰斗雪的英雄凯歌；面对举世震惊的汶川特大地震灾难，社会各界爱心涌流，情系巴蜀，全力支援，救困济难，为抗震救灾和灾后重建做出了突出贡献，展示了乐于奉献的精神风貌；面对国际金融危机的严峻挑战，全市上下坚定信心，迎难而上，有力应对，力克时艰，推动经济实现了高位增长，谱写了科学发展的壮丽诗篇。我们坚信，只要我们坚持弘扬自强不息、开拓创新、和衷共济、百折不挠的时代精神，就一定能为芜湖加速发展凝聚攻无不克、战无不胜的强大力量，创造出无愧于时代、无愧于历史的辉煌业绩！

各位代表，过去一年，面临的形势异常严峻，取得的成绩超过预想，回顾我们并肩走过的历程，倍感成绩来之不易。这是中共芜湖市委坚强领导的结果，是全市人民同舟共济、团结奋斗的结果。在此我谨代表市人民政府，向全体人大代表、政协委员，向全市广大工人、农民、知识分子、干部、驻芜解放军指战员、武警官兵和政法干警，向各民主党派、工商联、各人民团体和社会各界人士，向在我市创业的投资者、建设者和海内外友好人士，表示衷心的感谢和崇高的敬意！

在肯定成绩的同时，我们也清醒地看到前进道路上还有不少困难，工作中还有一些不足，主要是：经济总量不够大、产业结构不够优、整体经济抗风险能力不够强的矛盾长期存在，经济下行压力加大、企业经营困难、招商引资进展不够平衡的问题比较突出；节能减排任务十分艰巨，经济发展方式有待进一步转变；城市功能不够完善，管理水平有待进一步提升；财政保平衡压力加大，增收节支工作有待进一步强化；部分城乡居民生活相对困难，民生工作有待进一步加强；少数部门职能转变不够到位、工作作风不实、工作落实不力、办事效率不高，发展环境有待进一步优化。这些问题我们一定高度重视，采取有效措施加以解决。

二、2009年工作主要目标和任务

2009年是实施“十一五”规划至关重要的一年，可能是新世纪以来经济形势最为复杂严峻的一年，也是蕴含着重大机遇的一年。国际经济金融形势恶化对我国的影响不断加深，经济增长放缓、企业经营困难的状况正加快从沿海向内地、从中小企业向大企业、从出口行业向其它行业蔓延，保持增长的难度空前增大，对此我们必须保持清醒的认识。同时应该看到，促进发展也有很多有利因素，我国发展所处的重要战略机遇期没有改变，经济平稳较快发展的基本态势没有改变，我市厚积薄发、加速发展的阶段性特征没有改变；国家实施积极的财政政策和适度宽松的货币政策，将为我们的发展提供有利条件；国家实施汽车、钢铁、造船、纺织、有色金属、装备制造业和电子信息等九大行业的振兴计划，将为我市重点产业的发展提供难得机遇；我市区位条件优越，产业基础较好，人民加快发展意愿强烈，这些都为我们推动科学发展提供了坚实基础。面对挑战，我们别无选择，必须坚定信心，知难而进，把困难挑战估计得更充分一些，把应对措施准备得更周密一些，在逆水行舟中奋力拼搏，在应对挑战中把握机遇，努力夺取经济社会发展新胜利。

今年政府工作的总体要求是：认真贯彻党的十七大、十七届三中全会、中央和省经济工作会议精神，以邓小平理论和“三个代表”重要思想为指导，深入学习实践科学发展观，紧紧围绕保持经济平稳较快发展这条主线，大力推进自主创新、工业强市、三产兴市、创业富民、统筹发展、东向发展“六大战略”，着力扩大有效投入力保经济增长，着力推进自主创新转变发展方式，着力保障改善民生促进社会和谐，着力提高政府效能优化发展环境，努力推动科学发展、和谐发展、率先发展。

今年经济社会发展的主要预期目标是：全市生产总值比上年增长12%以上；财政收入与经济同步增长；全社会固定资产投资增长20%；城镇居民人均可支配收入增长8%，农民人均纯收入增长7%；新增城镇就业岗位4.2万个，城镇登记失业率控制在4.5%以内；人口出生率控制在11‰以内；万元地区生产总值综合能耗、二氧化硫和化学需氧量减排完成省政府下达任务。这个目标是指导性的，在当前严峻的宏观形势下，实现这样的目标并不容易，但经过努力是可以完成的，在实际工作中还要力争更高一些。实现上述目标，要突出做好以下工作：

（一）千方百计扩大有效投资

抢抓国家扩大内需政策机遇，着眼于打基础、管长远、增后劲，实施一批事关全局的重大项目，着力解决制约发展的瓶颈问题，增强经济社会发展的内在动力。

千方百计加快重大基础设施项目建设。推进宁安城际铁路、京福客运专线、皖赣客运专线、合芜杭客运专线四条快速铁路建设，推进淮南线、皖赣线、芜宣杭线、宁铜线四条铁路提速扩能电气化改造，加强以芜湖为枢纽的铁路站场、站房建设；加快芜申运河、芜雁高速建设进度；加快港口建设和岸线开发，推进芜湖长江二桥等项目前期工作，提升芜湖综合交通枢纽地位。积极争取上级专项资金支持，做好国债项目编报争取工作，切实加强国债项目建设和资金管理。

千方百计增加有效信贷投入。充分发挥银行信贷主渠道作用，强化银政企合作，加大优质企业、重点项目推介力度，引导银行机构加大信贷投放。充分发挥政府性担保典当机构、中小企业担保基金、中小企业贷款风险补偿基金和高科技风险投资公司的作用，引导金融机构支持“三农”和中小企业发展。加大争取外国政府和国际金融机构贷款工作力度，力争一批基础设施项目列入国家外贷计划。完成市建投公司10亿元债券发行。

千方百计扩大社会投资。持之以恒地将招商引资作为经济工作的重中之重，力争取得更大成效。抓住欧美日经济衰退、国际资本加速向我国转移的机遇，加大对欧美日地区世界500强等大企业的招商力度，力争一批企业来芜投资，形成产业发展新突破。抓住两岸关系趋好、台资加速向大陆转移的机遇，充分发挥社会中介组织作用，利用两岸直航港口城市优势，加大对台资的招商力度，力争我市台资企业形成集群。抓住东部沿海地区生产成本提高、产业加速向中西部地区转移的机遇，充分发挥国家产业梯度转移重点承接地优势，吸引一批企业来我市配套发展，延伸相关产业链条。把招商引资作为 “一把手”工程，各级各部门主要精力向招商工作集中，优秀人才向招商一线集中。完善统计考核激励机制，重奖招商有功人员，调动全社会招商引资积极性。加快推进经济技术开发区征地拆迁安置和基础设施建设，发挥龙头带动作用，力争在吸引大企业、大集团、外资企业上取得新进展。进一步提升各县区开发区产业承载能力和管理服务水平，做大做强特色鲜明、各具优势的产业集群。

（二）持续增强自主创新能力

增强自主创新能力，是调整经济结构、转变发展方式、实现可持续发展的战略性选择，也是提升整体经济素质、增强产业竞争力的必由之路。要全面推进合芜蚌自主创新综合配套改革试验区建设，大力发展技术水平较高、配套能力较强的产业集群，大力推进创新平台建设，努力在关键领域、关键产品、关键技术上形成突破。

打造产业创新发展优势。做大做强支柱产业。支持奇瑞公司创新、创业、创名牌，加快国家级节能环保汽车工程实验室建设，争取组建国家节能环保汽车工程技术中心，支持奇瑞公司推进轿车五厂、奇瑞量子汽车等项目建设，引进一批国内外重要汽车零部件配套商，提升汽车及零部件产业综合竞争能力。充分发挥海螺等大企业大集团人才资金优势，拓宽产业发展领域，推动鑫科、楚江等企业广泛采用先进技术，推进芜湖海螺三期、信义环保特种玻璃、精诚铜业高精度铜合金板带材、新兴铸管钢管坯生产线和径向锻造生产线等项目建设，扩大材料产业规模。推进美的日用家电基地等项目建设，支持美的、中达电子等企业加快发展，壮大电子电器产业。围绕精密加工、精密铸造、水泥成套设备、机电设备制造等产业，支持一批现有企业扩能升级，引入一批龙头企业提升水平，大力发展装备制造业。积极培育新兴产业。加快华强文化科技产业园等项目建设，推动文化创意、电子信息、节能环保、生物医药产业形成集群。利用先进适用技术改造提升传统产业。基本建成宝成鞋业、圣罗纺织等一批项目，加快芜湖电厂五期、响水涧抽水蓄能电站等项目建设，推进核电站等项目前期工作。大力扶持中小企业，完善发展非公有制经济的政策措施，在融资服务、财税扶持等方面搞好服务，努力形成全民竞相创业的良好局面。

加强创新服务体系建设。完善高新技术创业中心、软件园服务功能，加快汽车电子及关键零部件企业孵化器、动漫产业孵化中心等建设步伐，推进汽车电子产业园等产业发展基地建设，争取芜湖高新技术产业园区升级为国家级高新技术开发区。做大安徽长江产权交易所，打造科技成果转化服务平台。组建各类行业协会、商会，完善科技中介服务体系。加强政策引导，鼓励企业增加研发投入，建立各类研发机构。鼓励企业申报驰名商标、著名商标，及时将创新成果、核心技术申请专利，创建国家知识产权示范城市。支持企业建立知识产权入股、绩效工资等多元化分配机制，解决来芜人才生产生活后顾之忧，吸引各类人才来芜创业。实施“走出去”战略，支持奇瑞等有条件的企业在境外建立生产基地、营销网络和研发机构，吸引各类人才推动芜湖经济发展。

（三）全面推进农村改革发展

认真贯彻党的十七届三中全会精神，围绕稳粮、增收、强基础、重民生，创新农村体制机制，提高农业综合生产能力，推进基础设施和公共服务向农村延伸覆盖，推动城乡统筹发展。

深化农村改革。按照依法自愿有偿原则，鼓励农民以转包、出租、转让、股份合作等多种形式流转土地承包经营权，逐步建立土地承包经营权流转市场。抓好城镇建设用地增加与农村建设用地减少挂钩试点工作，加强土地开发复垦整理，提高土地综合利用能力。落实“一卡通”、“一线实”等措施，做好为民服务全程代理工作。出台鼓励农民进城的政策措施。完善征地补偿机制，健全被征地农民养老保障制度。

以工业理念大力发展都市型现代农业。加快农业结构调整步伐，加大市场化运作力度，促进超级水稻推广应用，发展优质粮油、苗木花卉、食用菌等特色产业，扩大产业规模。支持农业产业化龙头企业、农民专业合作社、执照农民、专业种养大户等市场主体实施规模经营。推进农村社会化服务组织建设，帮助农民实现与大市场的有效对接。发挥现代农业综合服务中心作用，加快建设芜湖气象中心，建立现代农业综合气象灾害预警预报系统，为农业发展提供服务支撑。认真落实国家强农惠农政策，提高农民生产积极性。深化集体林权制度配套改革，加大植树造林力度，完成营造林6.6万亩。

扎实推进新农村建设。紧紧抓住我市被列为省城乡一体化综合配套改革试验区机遇，按照“先行先试、率先突破”的要求，积极探索新农村建设新途径。完成三县中心镇农村新型社区居民点布点规划，加快编制村庄规划。推进“百村整治、十村示范”工程，抓好省、市示范村扩面工作，实施河渠清淤，整治农村生活环境。开展“百企联百村”活动，加大政府部门联系帮扶工作力度，推动社会资本参与新农村建设。加强农田水利基本建设，实施扬港、龙窝湖等泵站工程。改造湾石路等农村公路，完成芜南路快速通道三期拓宽改建和芜南供水、繁昌至铜陵段省道改建工程。

（四）深入实施三产兴市战略

充分发挥我市产业和区位交通优势，坚持规划引导、政策扶持，二、三产业联动，内引、外联并举，生产性、生活性服务业并重，着力提升服务业发展水平。

加快中石油油品码头、吉宝物流、安得物流、邮政物流等一批物流项目建设，推进现代物流与电子商务融合，大力发展第三方物流。实施城市商业网点规划，逐步整合现有市场，新建果品冻品海鲜批发市场、小商品批发市场等一批规模较大、档次较高的专业批发市场。加快推进美食街二期、滨江世茂商场、联盛商业广场等商业项目，积极培育大型综合超市、仓储式超市、连锁经营等新型商业业态，建设粮食产业园区和黄砂交易市场。落实好“家电下乡”等政策，新建和改造一批农村商品配送中心，提升“万村千乡”市场工程信息化水平，改善农村消费环境。优化出口产品结构，增加高附加值产品出口份额，扩大国际先进技术和设备进口。改进外汇管理，推动贸易投资便利化。促进房地产市场持续稳定健康发展，落实好促进住房消费的政策措施，发挥好住房公积金的支持作用，提高居民住房购买能力；综合运用多项措施，积极推进拆迁安置房、廉租住房、廉租公寓等保障性住房建设。大力发展旅游业，推进芜湖古城、马仁奇峰、丫山花海石林、生态体育公园等项目建设，完善景区配套设施。加快世茂、汉爵阳明二期等星级宾馆项目建设步伐，提高接待水平。办好第八届旅博会、第二届中国国际动漫产业博览交易会等专业特色会展。

完善地方金融组织体系。引进一批域外金融机构来芜发展，加快筹建三县农村合作银行。推动银行机构联合社会资本发起设立贷款公司、村镇银行等新型农村金融机构试点，规范小额贷款公司运营，引导民间资本服务经济发展。强化资本市场筹资工作，推动一批行业优势明显、主业突出、业绩良好的重点企业加快上市进程，争取有3户以上企业完成上市申报。规范信贷市场和债券市场信用评级，引导企业增强信用意识，帮助金融机构排忧解难，优化金融生态环境。

（五）着力加快城市建设步伐

坚持高起点规划、大手笔建设、精细化管理，打造综合环境一流、适宜居住兴业、比较优势明显的滨江山水园林旅游名城。

加强城乡规划工作。加快修编土地利用总体规划，严格执行土地利用年度计划。完善市域三县城乡总体规划、城市园林绿地规划、地下空间利用规划等一批规划，加强轨道交通和快速公交系统、城市空间景观等规划研究。

按照“东扩、南进、中提升”的思路推进城市大建设。加快建设城东新区，打造极具发展潜力的活力新区、园林新区和时尚新区，建成赤铸山东路、神山东路、神山南路，开工建设三环路跨青弋江大桥、经六路北延伸段、营盘山路东段、扁担河降堤工程。着力提升老城区服务功能，优化老城区交通环境，完成长江路和弋江路辅道、弋江路南延线、赭山路“刚改柔”和袁泽桥东幅桥工程，开工建设弋江路北延线和天门山路、吉和南路拓宽改造工程，加快镜湖新城、大砻坊旧城改造等项目建设步伐。完善城南路网体系，建成滨江南路二期、纬五路一期、花津南路延伸段、利民东路、峨山路辅道工程，开工建设三山疏港大道。投资1.6亿元推进三年大绿化工程，加强绿化美化工作督查。完成保兴垾综合整治、赭山公园西大门改扩建。加快区管道路、公园、老城区雨污分流改造项目建设。

推进生态城市建设。实施一批节能减排项目，淘汰小水泥等落后产能，推行清洁生产。建成朱家桥污水处理厂二期、城南污水处理厂一期、城北污水处理厂一期、三县污水处理厂，开工建设三山污水处理厂。落实节能减排工作责任制，健全统计监测考核体系。严格建设项目环境准入，落实环境保护“三同时”制度，完善市环境自动监控系统，强化对重点行业和重点企业的监管。继续开展整治违法排污企业保障群众身体健康环保专项行动，加强饮用水源保护和污染防治，严查环境违法行为。加强龙窝湖自然环境保护，争创2个国家级环境优美乡镇，争取国家级生态示范区建设试点通过省级验收。

创新城市管理体制机制。强化区级属地管理责任，实行市区联动考核，加强数字化城管系统建设，推动城市管理向精细化、常态化、数字化、法制化转变。深入开展第二阶段查处违法建设工作，健全长效管理机制。对城区重点路段、广场、公园和桥梁实施亮化工程，建设夜景亮化示范街。加强社区管理，实施小区环境综合整治，规范物业管理，开展第六届居委会换届直选，建立社会工作者队伍，创建城乡和谐社区。

（六）切实加强以改善民生为重点的社会建设

坚持将保障和改善民生作为政府工作的出发点和落脚点，在加快经济发展中，大力发展各项社会事业，着力实施民生工程，让230万江城人民共享改革发展成果。

做好就业和社会保障工作。认真落实就业再就业扶持政策，加强创业园建设，加大小额担保贷款力度，实施“创业扶持工程”，争创全国创业试点城市，以创业带动就业。加快技师学院建设，加强职业技能培训。发挥工会等社会组织作用，构建和谐劳动关系。完善养老保险市级统筹制度，做好养老保险省级统筹准备。推进失业保险市级统筹。健全医疗、工伤和生育保险制度。完善 “双基数”征缴机制，提高社保基金征缴率。加快金保工程建设，扩大劳动保障城域网覆盖面。推动非公有制企业建立住房公积金制度，维护职工权益。加快农村社会福利中心建设，发展慈善事业，基本建立覆盖城乡的社会救助和灾害应急救助体系。

发展教育、卫生、文化事业。完善教育均衡发展配套机制，加快普及高中阶段教育进程。加大教育投入，推进中小学标准化建设，健全义务教育经费保障机制，完善农村中小学现代远程教育工程，促进优质教育资源向农村辐射。完成市一中新校区主体工程，建设双语学校。创新职业教育发展模式，推进中职学校“重点学校、重点专业、重点实训基地”项目建设，开工建设芜湖工业学校新校区。提高高等教育质量，发展继续教育。打造皖江医疗中心。深化医药分开改革，逐步向县区公立医院和社区卫生机构延伸，全面实行药品购销网上交易、在线监控，争创全国医药卫生体制改革试点城市。建成市二院新建门诊医技楼和住院楼主体工程，加快市中医院新院区、市四院住院楼建设进度。整合医疗卫生资源，加强社区卫生服务机构规范化建设。开展医院管理年和平安医院创建活动。推进市博物馆建设，完善公共文化服务体系。加强县区镇街文化馆站建设，开展创建先进文化街道、社区试点工作，促进文化信息资源共享工程向基层覆盖。深化文化体制改革，完善用人机制，增强发展活力。搞好第二次全国经济普查和第三次全国文物普查。开展全民健身活动，办好第十一届市运会。

加强社会管理。加大争创全国文明城市工作力度，深入开展社会主义荣辱观教育和“做文明市民，创礼仪芜湖”活动，努力形成良好社会风尚。加强国家安全、国防教育和后备力量建设，开展创建“双拥合格单位”、“全省双拥模范县”活动。深入开展“五五”普法宣传教育，建立健全社区矫正体系，加强法律援助工作。支持工会、共青团、妇联等人民团体参与社会管理和公共服务，维护群众合法权益。加强社会养老服务机构建设，发展社会养老服务业。完善计划生育工作机制，改善技术服务条件，健全流动人口管理服务体系，稳定低生育水平。开展第二次全国土地大调查，加强国土资源执法监察，确保全市耕地保有量不减少、基本农田保护率不降低。巩固矿产资源开发秩序整治成果，加强矿山地质环境保护治理和地质灾害防治工作。创新信访工作机制，重点解决疑难复杂信访案件，严查违反信访纪律行为。严格落实安全生产责任制，强化安全生产基础工作和源头治理，突出抓好重点领域、重点单位的安全监管，防止重特大事故发生。完善公共应急体系，提高处置突发事件的能力。强化社会治安综合治理，加强治安防控体系建设，严厉打击各种犯罪活动，建设平安芜湖。

着力实施民生工程。按照“循序渐进、滚动发展、量力而行、尽力而为”的原则，实施32项民生工程。完善城乡居民最低生活保障制度；完善农村“五保户”供养制度；完善城镇养老保障机制；建立覆盖全市的新型农村合作医疗制度；完善城镇居民基本医疗保障制度；逐步提高城乡医疗救助水平；建立重大传染病病人医疗救治及生活救助保障机制；推进城乡卫生服务体系建设；全面实施城乡义务教育经费保障机制改革；推进农村饮水安全工程建设；实施农村公路“村村通”工程；完善农村计划生育家庭奖励扶助制度；实施广播电视“村村通”工程；建立高校和中职学校家庭经济困难学生资助制度；完善大中型水库移民后期扶持政策；建立城市低收入家庭住房困难保障机制；实施贫困白内障患者复明工程；实施“农家书屋”工程；实施乡镇综合文化站建设工程；实施农民体育健身工程；建立政策性农业保险制度；加快农村五保供养服务机构建设；实施重度残疾人生活救助；实施新型农民培训工程；实施农民工技能培训工程；实施农村沼气建设工程；实施病险水库除险加固工程；提高妇女儿童健康水平；实施就业扶持工程；实施市区棚户区拆迁改造工程；实施食品药品安全工程；实施市区菜市场改造工程。

三、建设高效廉洁的民本政府

以推进管理创新为重点，以提高行政效能为突破口，着力加强政府自身建设，不断提高服务人民、服务社会的能力和水平。

大力推动思想解放，营造科学发展的浓厚氛围。深入开展学习实践科学发展观活动，着力转变阻碍科学发展的思想观念，着力破除制约科学发展的体制机制。努力学习新知识，研究新形势，增长新本领，提高领导科学发展的能力。开展“企业、社区、乡村”大调研活动，问政于民，问需于民，问计于民。树立科学理念，增强忧患意识，建设创新型城市。

大力推进依法行政，建设法治政府。自觉接受人大及其常委会监督和政协民主监督，认真听取各民主党派、工商联、无党派人士及各人民团体意见，广泛接受社会监督，不断改进政府工作。认真办理人大议案、代表建议和政协建议案、提案。积极稳妥推进新一轮政府机构改革，进一步扩大县区政府经济社会管理权限，深化乡镇机构改革。继续推进行政审批制度改革，清理规范性文件，把工作重心从事前审批转向事后服务和监管。深化行政执法体制改革，探索扩大相对集中行政处罚权工作，严格执行行政执法责任制和过错责任追究制。

大力加强政风建设，建设服务政府。将主要精力用于抓落实、促发展上，用于抓热点、破难题上，完善经济运行调度制度，紧紧围绕企业发展、项目建设这个重心，强化“一线工作法”，深入企业、深入现场，协调服务，解决难题，帮助企业渡过难关。精简会议、文件，改进会风、文风，推行“马上办”、“现场办”、“限时办”工作制度，优化工作流程，促进机关运转更加高效规范。加强电子政务建设，实现各级政府部门网上办公。完善行政审批“两个集中”工作机制，扩大并联审批范围，规范运行行政审批电子监察系统，提高审批效率和工作透明度。探索建立科学统一的绩效考核指标体系，完善重点工作跟踪落实机制，形成科学的政府督办机制，提高工作落实力和执行力。完善各类公开办事制度，扩大政务公开渠道和范围，对群众关心、社会关注的政府信息，做到全面公开；涉及公权力大、公益性强、公众关注度高的公共政策和重要事项，做到及时公开，让权力在阳光下规范运行。

大力加强廉政建设，建设廉洁政府。按照《建立健全惩治和预防腐败体系2008－2012年工作规划》的要求，坚持标本兼治、综合治理、惩防并举、注重预防的方针，在坚决惩治腐败的同时，更加注重治本，更加注重预防，更加注重制度建设。规范项目招标、土地出让、政府采购、拆迁安置等工作，严肃查处违法违纪行为。加大行政问责力度，充分发挥监察、审计作用，强化政府内部监督。始终保持艰苦奋斗、勤俭节约的优良传统，牢固树立过“紧日子”思想，坚决取消不合理、不必要的支出，行政性开支实行零增长。严格执行廉洁从政规定，坚决杜绝奢侈浪费现象。

各位代表，新的一年，新的征程，激越豪迈的号角已经吹响。在前进的道路上，困难和挑战考验着我们，机遇和希望召唤着我们，责任和使命鞭策着我们。让我们更加紧密地团结起来，在中共芜湖市委的坚强领导下，坚定信心，解放思想，勇于创新，开拓奋进，书写加快发展的新篇章，创造更加美好的新生活，以优异的成绩迎接新中国成立60周年！
